# One‐Pot Gram‐Scale Synthesis of Cynandione A and Detailed Mechanistic Insights into its Regioselectivity

**DOI:** 10.1002/open.202500001

**Published:** 2025-03-26

**Authors:** Jun Sung Kang, Jin Yi Jung, Chan Jae Cho, Seoungwoo Kang, Yeonjoon Kim, Jae Hyun Kim

**Affiliations:** ^1^ Department of Global Innovative Drugs Chung-Ang University Seoul 06974 Republic of Korea; ^2^ College of Pharmacy Chung-Ang University Seoul 06974 Republic of Korea; ^3^ Department of Chemistry Pukyong National University Busan 48513 Republic of Korea

**Keywords:** Biaryls, Density functional calculations, Quinones, Regioselectivity, Total synthesis

## Abstract

Cynandione A, a natural biaryl product with a bis‐acetophenone structure isolated from *Cynanchi wilfordii* Radix, has garnered considerable attention because of its diverse biological activities, including anti‐inflammatory, neuroprotective, antioxidant, and adipogenic effects. However, the limited natural and commercial availability of cynandione A impedes large‐scale evaluations, particularly in vivo studies. Herein, we report a one‐pot gram‐scale synthesis of cynandione A. The key step of this synthesis is a regioselective conjugate addition of acetyl bisphenol to a benzoquinone substrate, which has been mechanistically explored using density functional theory (DFT) calculations. The significant energetic differences at the transition states among the possible pathways indicate the C3 position of the acetyl bisphenol nucleophile as the exclusive site of addition. Through careful optimization, the one‐pot synthesis affords cynandione A in a gram scale with a high yield. This method presents a practical, and scalable approach for synthesizing cynandione A with excellent pot economy.

## Graphical abstract

An improved one‐pot, gram‐scale synthesis of cynandione A, a key natural product from **Cynanchi wilfordii Radix** with diverse biological activities, has been developed. DFT calculations clarified the regioselectivity of the arylation step in the benzoquinone, revealing that the C3 position of the nucleophile is highly favored due to both kinetic and thermodynamic factors.

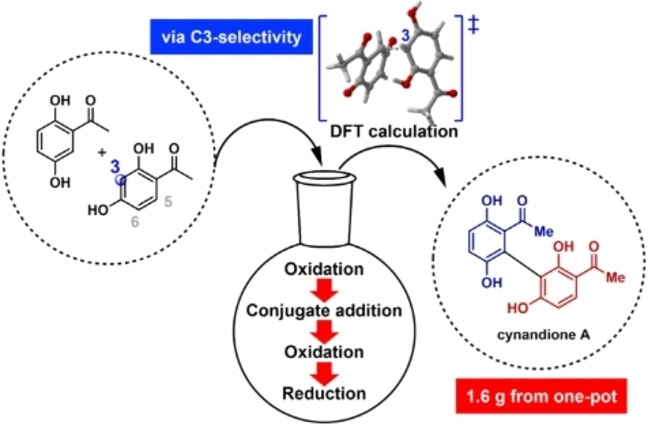

## Introduction

**Scheme 1:**
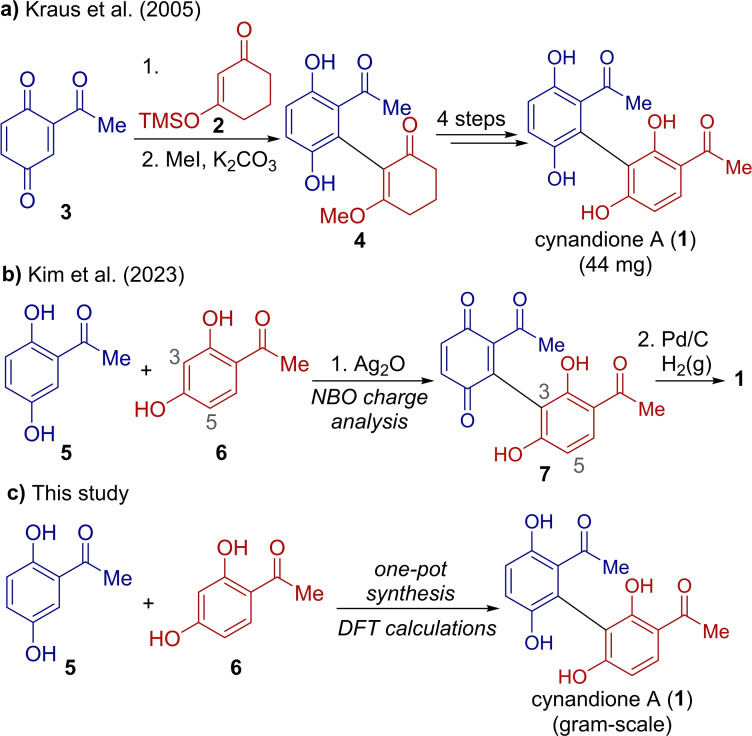
Preivous syntheses and present one‐pot synthetic strategy on cynandione A (**1**).

Cynandione A (**1**) is a natural biaryl product with a bis‐acetophenone structure and diverse biological activities (Scheme 1).[^1^, ^2^] It is the primary constituent of *Cynanchi wilfordii* Radix, which is a traditional remedy in East Asian medicine used to treat various medical conditions, including insomnia, anxiety, anemia, senescence, and other geriatric diseases.[^3^, ^4^, ^5^, ^6^, ^7^] In addition, research on **1** has gained traction owing to its potential therapeutic applicability and intriguing biological activities, including anti‐ inflammatory, neuroprotective, antioxidant, and adipogenesis activities.[^8^, ^9^, ^10^, ^11^, ^12^, ^13^, ^14^, ^15^, ^16^, ^17^, ^18^, ^19^]

The isolation of **1** from 500 mg of the EtOAc extract, part of the 7 g extract obtained from 300 g of *Cynanchi wilfordii* Radix, yielded 9.6 mg of **1**.^[18]^ In addition to its natural sources, **1** is commercially available, with ten suppliers listed on SciFinder (December 2024). For instance, although its availability is marked as “limited or intermittent availability”, Accela offers 250 mg of cynandione A for $1900 ($7.6/mg). TargetMol, which maintains its stock, lists this compound at $518/mg. While the availability of multiple commercial suppliers highlights the scientific community‘s interest in **1** and its significance, the small‐scale availability of **1** from natural and commercial sources, typically in milligrams, is insufficient for extensive investigations into its biological activities, particularly for evaluations in animal models. Therefore, developing a method to obtain large quantities of **1** is both highly necessary and challenging.

The first synthesis of compound **1** was reported by Kraus et al. in 2005 (Scheme 1a).^[20]^ The synthesis involved the nucleophilic addition of the silyl enol ether of cyclohexane‐1,3‐dione **2** to acetylbenzoquinone **3**, yielding **4**, followed by additional steps including Friedel‐Crafts acylation and deprotection. Although **1** was synthesized in six steps, the modest yield of 44 mg was insufficient to support thorough biological evaluation.

More recently, our group has achieved a gram‐scale synthesis of **1** through the regioselective conjugate addition of acetyl bisphenol **6** to a benzoquinone **3** derived from acetyl bisphenol **5**, followed by a subsequent reduction of **7** (Scheme 1b).^[21]^ The regioselectivity in the conjugate addition step was explained using natural bond orbital (NBO) charge analysis of acetyl bisphenol **6**. However, further improvements, such as reducing the number of pots, could enhance the efficiency of the synthesis. For large‐scale synthesis, minimizing the number of pots and enhancing the “pot economy” are crucial.[^22^, ^23^, ^24^, ^25^] Reducing the number of pots streamlines the synthesis process by cutting down on purification steps, additional operations, and solvent use, ultimately saving both time and cost. As a result, pot‐economic synthesis offers both cost‐effectiveness and environmental benefits.[^22^, ^23^, ^24^] Particularly, one‐pot processes streamline chemical synthesis by facilitating multiple sequential reactions within a single vessel. Although limited, efforts are underway to develop one‐pot synthesis methods for biologically relevant natural products.[^26^, ^27^, ^28^] This approach minimizes the need for intermediate isolation and purification, significantly enhancing efficiency while conserving time and resources. By reducing solvent use and waste generation, one‐pot strategies also align with the principles of green chemistry, making them a sustainable and practical choice for modern organic synthesis.[^29^, ^30^, ^31^]

In the previous study, NBO charge analysis of acetyl bisphenol **6** was used to explain the regioselectivity in the conjugate addition step.^[21]^ However, this analysis is insufficient to fully determine the underlying mechanism, as the charge difference between C3 (−0.370) and C5 (−0.336) is marginal (only 0.034), which does not adequately account for the observed high regioselectivity. Therefore, more precise methods are needed to thoroughly rationalize this selectivity and provide a robust explanation for the regioselectivity in the conjugate addition.

The present study introduces a one‐pot, gram‐scale synthesis of **1**, offering an efficient and practical method for producing large quantities of **1** (Scheme 1c). Additionally, detailed DFT calculations that provide insights into the exceptional regioselectivity observed in the conjugate addition reaction are presented.

## Results and Discussion

**Scheme 2:**
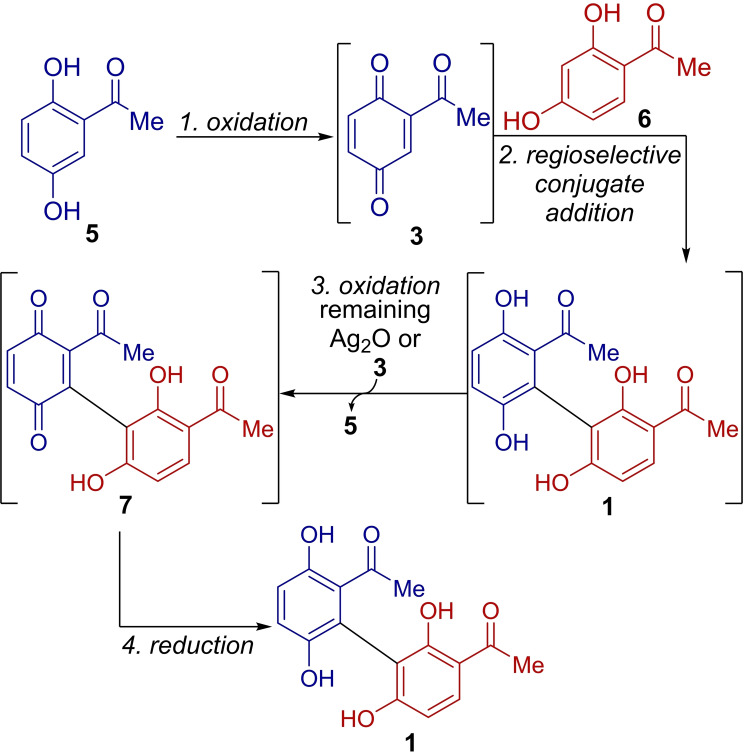
Sequential chemical transformations involved in the one‐pot synthesis of **1**.

The one‐pot synthesis of **1** involves four sequential chemical transformations within a single pot (Scheme 2). First, **5** is oxidized to its corresponding benzoquinone **3**. Second, the regioselective conjugate addition of **6** to benzoquinone **3** generates **P‐3**, which readily undergoes aromatization to produce **1** (vide infra). Third, **1** is inevitably oxidized to its benzoquinone form, compound **7**, either by a co‐existing oxidant (Ag_2_O) or compound **3**. Finally, benzoquinone **7** is reduced to **1** under reducing conditions. The rapid *in situ* oxidation of **1** to **7** in the third step is unavoidable because of the inherent antioxidant activity of **1**, which was previously revealed by the redox reaction between **3** and **1** to yield **5** and **7**.^[21]^ To ensure complete consumption of the starting materials **5** and **6**, an excess amount of oxidant is required, leading to the formation of compound **7**.

Theoretically, 2.0 equiv. of oxidants is required: 1.0 equiv. for the oxidation of **5** to its benzoquinone and 1.0 equiv. for the oxidation of **1** or regenerated **5**, produced via the redox reaction between the benzoquinone **3** and **1**. In contrast to the earlier synthesis, in which 2.5 equiv. of Ag_2_O was used, in the present study, we attempted to minimize the amount of this oxidizing agent used in the synthesis owing to its high costs. The crude ^1^H NMR (nuclear magnetic resonance) spectroscopy analysis indicated that the amount of Ag_2_O could be reduced to 2.1 equiv. to produce **7** as the only identifiable product without generating notable side products or leaving unreacted starting materials **5** or **6** (see ESI for details).

Notably, the success of the proposed one‐pot synthesis highly depends on identifying reduction conditions that are compatible with the reaction mixture, particularly in the presence of excess oxidants and byproducts from the oxidation step. To address this, we explored various reduction conditions that could be applied in the same pot, ensuring compatibility with both the reagents and solvents. Additionally, chemoselectivity was carefully considered to prevent the reduction of the acetyl groups.

After the *in situ* generation of the intermediate **7** from the reaction of **5** and **6** using 2.1 equiv. of Ag_2_O, various reduction conditions were subsequently applied directly in the same pot (Table 1). The previously employed reduction conditions on isolated **7**, using Pd/C and H_2_ gas, proved incompatible with the one‐pot reaction. Most of intermediate **7** remained unreacted, and only a trace amount of the desired compound **1** was detected. The addition of MeOH as a co‐solvent had no significant impact, as the yield of **1** remained negligible both in the presence and absence of MeOH (entries 1 and 2).

**Table 1:** Optimization of the reduction conditions of **7** for the one‐pot synthesis of 1.^[a]^

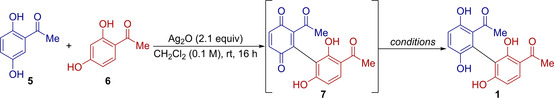					
Entry	Reducing reagent (equiv)	Solvent^[b]^	Temperature	Time (h)	Yield of **1** ^[c]^ (%)
1^[d]^	Pd/C, H_2_(g)	CH_2_Cl_2_ (0.1 M)	RT	13	trace
2^[d]^	Pd/C, H_2_(g)	CH_2_Cl_2_/MeOH (2 : 1, 0.066 M)	RT	13	trace
3	Na_2_S_2_O_4_ (2.0)	CH_2_Cl_2_/H_2_O (1 : 3, 0.025 M)	RT	1	11
4	NaBH_4_ (1.2)	CH_2_Cl_2_/MeOH (1 : 1, 0.05 M)	0 °C	0.5	21
5	Zn (2.0)	CH_2_Cl_2_/AcOH/H_2_O (2 : 1 : 1, 0.05 M)	RT	2	59
6	Zn (1.0)	CH_2_Cl_2_/AcOH/H_2_O (2 : 1 : 1, 0.05 M)	RT	2	79
7	Zn (1.0)	CH_2_Cl_2_/AcOH/H_2_O (10 : 6 : 3, 0.05 M)	RT	24	66

[a] Reaction conditions: **5** (1 mmol), **6** (1 mmol), Ag_2_O (2.1 mmol); reduction conditions were applied after adding the co‐solvents under N_2_ atmosphere. [b] Ratio in parentheses indicates volume ratio. [c] Yield of the isolated product. [d] 10 wt% Pd/C was used.

Using sodium dithionite (Na_2_S_2_O_4_), a reductant commonly used for benzoquinone moieties,^[32]^ with water as a co‐solvent, intermediate **7** was completely consumed. However, the reaction resulted in a complex mixture and the yield of the desired compound **1** was only 11 % (entry 3). Furthermore, sodium borohydride (NaBH_4_)^[33]^ reduced the acetyl carbonyl groups, leading to poor chemoselectivity. Even with precise adjustment of the NaBH_4_ amount (up to 1.2 equiv), the simultaneous reduction of the acetyl carbonyl groups could not be avoided, leading to a low yield (21 %) of compound **1** (entry 4).

Notably, using zinc (2.0 equiv) as the reducing agent, with water and acetic acid (AcOH) as co‐solvents, the one‐pot synthesis of compound **1** was achieved with a yield of 59 % (entry 5).^[34]^ Encouraged by this result, we further optimized the reaction by reducing the amount of zinc. This adjustment, using just 1.0 equivalent of zinc, improved the yield to 79 % (entry 6).

**Scheme 3:**
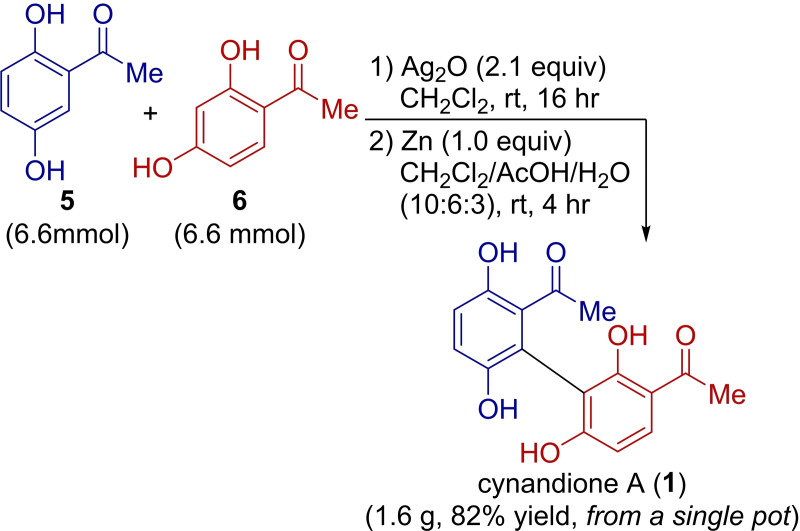
One‐pot gram‐scale synthesis of cynandione A (**1**).

With the successful identification of one‐pot compatible reduction conditions, the one‐pot synthesis was subsequently scaled up (Scheme 3). Fine‐tuning of the reaction conditions lead to the use of CH_2_Cl_2_/AcOH/H_2_O (10 : 6 : 3) co‐solvent system, resulting in a significant improvement (see ESI for details). In addition, special caution was necessary during the purification of cynandione A (**1**), as it has a tendency to dimerize to form various byproducts, including cynanchone A and cynandiones B and C.^[35]^ Thus, to prevent its dimerization and increase the yield of **1**, we used a short‐column chromatography setup (diameter: 3 cm; length: 30 cm) filled with 50 g of silica gel for the purification of **1**. Overall, we successfully obtained cynandione A (**1**, 1.6 g), with a yield of 82 %, from the one‐pot 6.6‐mmol‐scale reaction. When the reaction was carried out sequentially in a two‐pot process, compound **1** was obtained with a 79 % yield (see ESI for details), highlighting the efficiency of one‐pot synthesis in both pot economy and overall product yield. The optimized one‐pot conditions, specifically the fine‐tuned co‐solvent system, proved to be less effective on a 1 mmol scale, resulting in a lower yield of compound **1** and an extended reaction time (Table 1, entry 7).

Throughout the experiments, the conjugate addition of **6** to **3** consistently yielded only the desired product, with no other regioisomeric compounds formed. For compound **3**, the C3 position is more electrophilic than the C5 or C6 positions because of the presence of the electron‐withdrawing acetyl group at C2. Although previous NBO charge analysis for compound **6** indicated that the C5 position is slightly more electronegative than the C3 position,^[21]^ the difference is marginal and does not explain the observed extreme regioselectivity. Thus, to analyze the mechanism underlying this extreme regioselectivity, we calculated the reaction energetics and compared them for the three conjugate addition pathways at C3, C5, and C6 in compound **6**.

**Figure 1:**
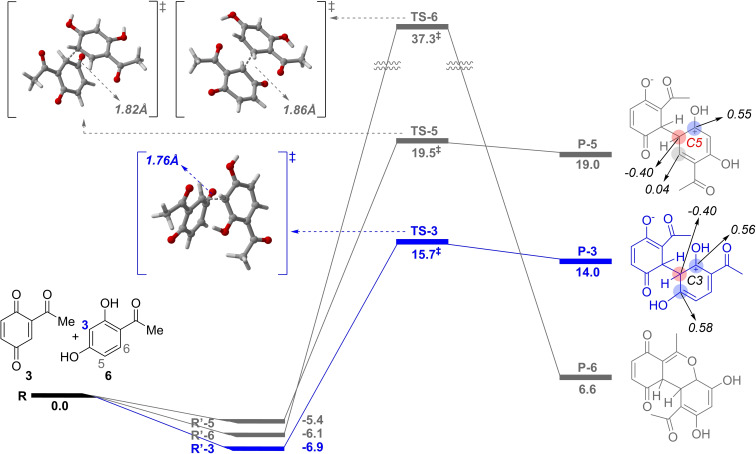
Proposed mechanisms and calculated energy diagrams for the three possible reaction pathways of the conjugation addition of **3** to **6**. Zero‐point corrected energies (kcal/mol) calculated at the M06‐2X/def2‐TZVP level of theory using the solvation model based on density of CH_2_Cl_2_ have been presented here. NBO charges are observed for some carbon atoms in **P‐3** and **P‐5**. The three‐dimensional geometries of the transition states, with the distance between the two carbons (where the C−C bond is formed during the reaction), are shown in the upper left.

Figure 1 illustrates the calculated energy diagrams for the three reaction pathways. Initially, the distance between **6** and **3** is infinite (**R**), and the relative energy is set to 0.0. Next, **6** and **3** are properly aligned to induce conjugate addition. Specifically, a pair of carbon atoms should face each other: C3, C5, or C6 in **6** and the unsubstituted carbon ortho to the acetyl group in **3**. Such aligned structures were found by the intrinsic reaction coordinate calculations. The aligned reactant states for the addition at C3, C5, and C6 are denoted as **R′‐3**, **R′‐5**, and **R′‐6**, with relative energies of −6.9, −5.4, and −6.1 kcal/mol, respectively. The energy differences between the initial reactant states do not exceed 1.5 kcal/mol, whereas the transition‐state (TS) energies show significant differences. The TSs clearly indicate the regioselectivity to C3; for the conjugate addition at C3, the TS (**TS‐3**) shows the lowest relative energy of 15.7 kcal/mol. Meanwhile, the addition at C5 and C6 should pass through the TSs with the relative energies of 19.5 (**TS‐5**) and 37.3 kcal/mol (**TS‐6**), respectively. **TS‐5** is 3.8 kcal/mol higher than **TS‐3**, while an excessive energy of 21.6 kcal/mol is required for the reaction to proceed through **TS‐6** instead of **TS‐3**, making the reaction at C6 virtually impossible.

The fraction of reactants passing through each addition pathway was determined from the Boltzmann‐weighted average of the TS energies. At room temperature, 99.8 % of the reactants react via **TS‐3**, while only 0.2 % and 0.0 % undergo addition at C5 and C6 through **TS‐5** and **TS‐6**, respectively. Additionally, a comparison of the distances between the two carbons involved in bond formation in the TSs, shown in the upper‐left part of Figure 1, reveals C−C bond distances of 1.76 Å, 1.82 Å, and 1.86 Å for **TS‐3**, **TS‐5**, and **TS‐6**, respectively. The shortest bond distance in **TS‐3** reflects the strongest attractive interaction between the reactants, resulting in the highest stabilization of the TS and a strong regio‐preference for C3.

The conjugate additions at C3, C5, and C6 result in the products **P‐3**, **P‐5**, and **P‐6**, with relative energies of 14.0, 19.0, and 6.6 kcal/mol, respectively. **P‐3** has an energy 5.0 kcal/mol lower than **P‐5**, demonstrating that the conjugate addition at C3 is both kinetically and thermodynamically more favorable than at C5. The lower energy of **P‐3** is due to the presence of −OH groups on both carbons adjacent to C3, while only one carbon adjacent to C5 in **P‐5** has an −OH group.

This difference results in varying NBO charges: the atomic charge of C3 in **P‐3** is −0.40, with adjacent carbons at 0.56 and 0.58. These alternating positive and negative charges stabilize **P‐3**. In contrast, C5 in **P‐5** shows an NBO charge of −0.40, but the adjacent carbons have charges of 0.55 and 0.04. The less positive charge of 0.04 contributes to the lower stability of **P‐5** compared to **P‐3**.

In contrast, conjugate addition at C6 results in ring formation, leading to a product with three fused rings (**P‐6**). This ring formation stabilizes the polarization of positive and negative charges within a molecule. While **P‐3** and **P‐5** contain atoms with formal positive or negative charges, all formal charges in **P‐6** are neutral, resulting in its lowest relative energy (6.6 kcal/mol) compared to **P‐3** and **P‐5**. However, the formation of **P‐6** requires overcoming a very high energy barrier at **TS‐6** (37.3 kcal/mol), making its formation almost negligible (~0.0 %). These computational results clearly demonstrate the high regioselectivity in the conjugate addition step and provide mechanistic insights into the facile synthesis of **1**.

## Conclusions

In conclusion, we successfully developed an efficient one‐pot, gram‐scale synthesis of cynandione A (**1**). The key to the success was the identification of one‐pot and gram‐scale compatible reduction conditions. The regioselectivity of the key conjugate addition step was thoroughly examined using DFT calculations, which revealed that the C3 position of the nucleophile is highly favored due to both kinetic and thermodynamic factors. This optimized synthesis not only achieves high yields but also improves pot and cost efficiency. The scalable approach significantly enhances the availability of cynandione A (**1**) for further investigations, particularly for in vivo applications, providing a valuable tool for exploring its therapeutic potential.

## Experimental Section

### General methods

All the chemicals were of reagent grade and were used as purchased. All the reactions were induced under an inert atmosphere of dry nitrogen using distilled dry solvents. The reactions were monitored using thin‐layer chromatography (TLC) with silica gel 60 F‐254 plates (40 mm ×10 mm). The compounds on the TLC plates were visualized under ultraviolet light and sprayed with either potassium permanganate or anisaldehyde solutions. Flash column chromatography was performed using silica gel 60 (230–400 mesh). The melting points were measured using a Buchi M‐560 melting point apparatus without correction. ^1^H and ^13^C nuclear magnetic resonance (NMR) spectra were recorded on a JEOL 600 MHz Fourier transform spectrometer at ambient temperature. The chemical shifts were reported in ppm (δ) units relative to the reference peak of the solvent (^1^H NMR: DMSO‐*d_6_* (2.50 ppm); ^13^C NMR: DMSO‐*d_6_* (40.00 ppm)). The NMR peak multiplicities were designated as s (singlet), d (doublet), t (triplet), m (multiplet), dd (doublet of doublets), dt (doublet of triplets), td (triplet of doublets), and br (broad signal).

### One‐Pot Gram‐Scale Synthesis of Cynandione A

Ag_2_O (3.2 g, 2.1 equiv) was added to a solution of **5** (1.0 g, 6.57 mmol, 1.0 equiv) and **6** (1.0 g, 6.57 mmol, 1.0 equiv) in CH_2_Cl_2_ (50 mL). The mixture was stirred at room temperature for 16 h under a nitrogen atmosphere. Next, AcOH (30 mL), water (15 mL), and zinc (430 mg, 1.0 equiv) were added to the crude reaction mixture, which was then stirred at room temperature for 4 h. The mixture was filtered through Celite, and the filtrate was diluted and extracted with EtOAc twice. The organic layer was washed with water and brine and subsequently dried over MgSO_4_. The solvent was removed under reduced pressures, and the residue was purified by flash chromatography on silica gel (hexane/acetone, 2 : 1) to yield **1** (1.6 g, 82 %). All spectroscopic data matched previously reported values. ^1^H NMR (600 MHz, DMSO‐*d*_6_) δ 10.31 (s, 1H), 9.31 (s, 1H), 8.52 (s, 1H), 7.71 (d, *J*=9.0 Hz, 1H), 6.74 (d, *J*=8.7 Hz, 1H), 6.69 (d, *J*=8.7 Hz, 1H), 6.44 (d, *J*=8.9 Hz, 1H), 2.54 (s, 3H), 2.21 (s, 3H); ^13^C{^1^H} NMR (150 MHz, DMSO‐*D*_6_) δ 203.15, 203.06, 162.50, 162.29, 147.95, 146.93, 132.21, 130.20, 118.10, 117.34, 115.75, 112.32, 111.49, 107.48, 30.70, 26.19.

## Conflict of Interests

The authors declare no conflict of interest.

## Supporting information

As a service to our authors and readers, this journal provides supporting information supplied by the authors. Such materials are peer reviewed and may be re‐organized for online delivery, but are not copy‐edited or typeset. Technical support issues arising from supporting information (other than missing files) should be addressed to the authors.

Supporting Information

## Data Availability

The data that support the findings of this study are available in the supplementary material of this article.
