# Supplementary material for: One‐Pot Gram‐Scale Synthesis of Cynandione A and Detailed Mechanistic Insights into its Regioselectivity
Source: ChemistryOpen. 2025 Mar 26;14(8):e202500001. doi: 10.1002/open.202500001 (PMC12368894; doi:10.1002/open.202500001)
Supplement: Supplementary file 1 — Supporting Information [file OPEN-14-e202500001-s001.pdf]

ChemistryOpen

Supporting Information

One-Pot Gram-Scale Synthesis of Cynandione A and Detailed Mechanistic Insights into its Regioselectivity

Jun Sung Kang, Jin Yi Jung, Chan Jae Cho, Seoungwoo Kang, Yeonjoon Kim,* and Jae Hyun Kim*

Supporting Information

One-Pot Gram-Scale Synthesis of Cynandione A and Detailed Mechanistic Insights into its Regioselectivity

Jun Sung Kang,[a†] Jin Yi Jung,[a†] Chan Jae Cho,[a] Seoungwoo Kang,[b] Yeonjoon Kim*[c] and Jae Hyun Kim*[a,b]

[a]*Department of Global Innovative Drugs, Chung-Ang University, Seoul 06974, Republic of Korea.*

[b]*College of Pharmacy, Chung-Ang University, Seoul 06974, Republic of Korea.*

[c]*Department of Chemistry, Pukyong National University, Busan 48513, Republic of Korea*

yeonjoonkim@pknu.ac.kr

jaehyunkim@cau.ac.kr

Table of Contents

1. General methods·····S3

2. One-pot gram-scale synthesis of cynandione A (1)·····S4–S7

3. DFT calculations·····S8–S15

4. References·····S16

5. Copies of spectra (^{1}H NMR, ^{13}C NMR)·····S17–S18

1. General methods

All the chemicals were of reagent grade and were used as purchased. All the reactions were induced under an inert atmosphere of dry nitrogen using distilled dry solvents. The reactions were monitored using thin-layer chromatography (TLC) with silica gel 60 F-254 plates (40 mm × 10 mm). The compounds on the TLC plates were visualized under ultraviolet light and sprayed with either potassium permanganate or anisaldehyde solutions. Flash column chromatography was performed using silica gel 60 (230–400 mesh). The melting points were measured using a Buchi M-560 melting point apparatus without correction. ^{1}H and ^{13}C nuclear magnetic resonance (NMR) spectra were recorded on a JEOL 600 MHz Fourier transform spectrometer at ambient temperature. The chemical shifts were reported in ppm (δ) units relative to the reference peak of the solvent (^{1}H NMR: DMSO-d_6 (2.50 ppm); ^{13}C NMR: DMSO-d_6 (40.00 ppm)). The NMR peak multiplicities were designated as s (singlet), d (doublet), t (triplet), m (multiplet), dd (doublet of doublets), dt (doublet of triplets), td (triplet of doublets), and br (broad signal).

2. One-pot gram-scale synthesis of cynandione A (1)

• Optimization of the synthesis of intermediate **7** using reduced amount of Ag_2O (2.1 equiv).

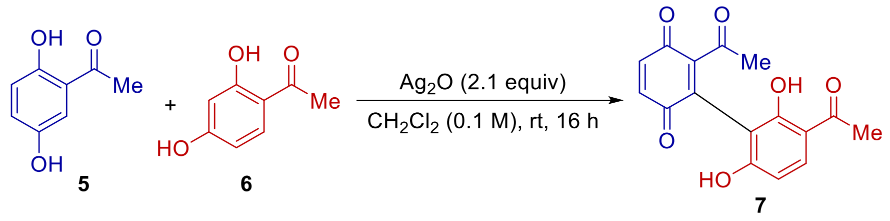

Ag_2O (489 mg, 2.1 equiv) was added to a solution of **5** (153 mg, 1.0 mmol, 1.0 equiv) and **6** (153 mg, 1.0 mmol, 1.0 equiv) in CH_2Cl_2 (10 mL) at room temperature, and the resulting mixture was stirred at the same temperature for 16 h under a nitrogen atmosphere. The mixture was then filtered over Celite and concentrated under a reduced pressure. Compound **7** was the only identifiable product, and the amounts of the side products or unreacted starting materials **5** and **6** were negligible, as confirmed by the crude ^{1}H NMR analysis. Compound **7**, crude ^{1}H NMR (600 MHz, DMSO-d_6) δ 11.05 (s, 1H), 7.85 (d, J = 8.9 Hz, 1H), 7.07 (d, J = 10.2 Hz, 1H), 7.00 (d, J = 10.2 Hz, 1H), 6.49 (d, J = 8.9 Hz, 1H), 2.56 (s, 3H), 2.24 (s, 3H). For spectra, see page S17.

• Optimizations of the reduction conditions of **7** in the one-pot synthesis method (Table 1 in the main text).

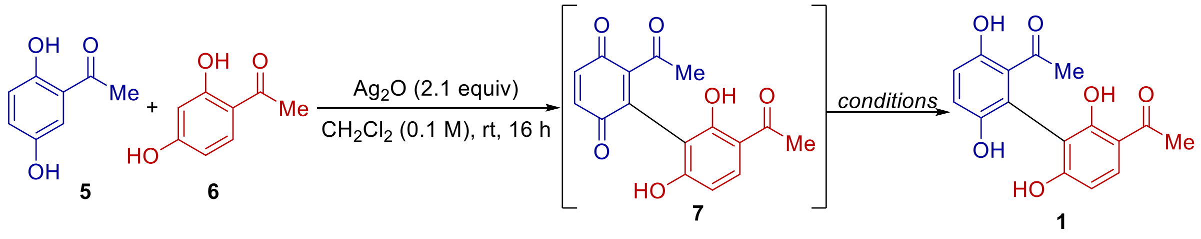

Ag_2O (489 mg, 2.1 equiv) was added to a solution of **5** (153 mg, 1.0 mmol, 1.0 equiv) and **6** (153 mg, 1.0 mmol, 1.0 equiv) in CH_2Cl_2 (10 mL) at room temperature. The mixture was stirred for 16 h under a

nitrogen atmosphere. The following reduction conditions were applied to the crude reaction mixture of **7** in the same pot.

Entry 1: Pd/C (30 mg, 10 wt%) was added to the crude reaction mixture of **7**, and then bubbled with H_2 gas for 30 min, and stirred for 13 h. Subsequently, the mixture was filtered through a Celite pad, and the filtrate was concentrated. Only trace amounts of product **1** were detected.

Entry 2: The same procedure as that in Entry 1 was followed by the addition of MeOH (5 mL) to the reaction mixture before introducing Pd/C. After 13 h of stirring under an H_2 atmosphere, only trace amounts of product **1** were detected.

Entry 3: H_2O (30 mL) and $Na_2S_2O_4$ (350 mg, 2.0 equiv) were added to the crude reaction mixture of **7**. After stirring for 1 h, the reaction mixture was quenched with a saturated NH_4Cl solution, filtered through Celite, and extracted twice with EtOAc. The combined organic layers were washed with brine, dried over $MgSO_4$, and concentrated under reduced pressures. The crude product was purified using flash chromatography on silica gel (hexane/acetone, 2:1) to yield **1** (32 mg, 11%).

Entry 4: MeOH (10 mL) was added to the crude reaction mixture of **7**, followed by the addition of $NaBH_4$ (34 mg, 1.2 equiv) in portions at 0 °C. The reaction mixture was stirred for 30 min at 0 °C. The reaction was quenched with water, extracted twice with EtOAc, washed with brine, dried over $MgSO_4$, and concentrated under reduced pressures. The residue was purified using flash chromatography on silica gel (hexane/acetone, 2:1) to yield **1** (63 mg, 21%).

Entries 5 and 6: AcOH (5 mL) and water (5 mL) were added to the crude reaction mixture of **7**, followed by the addition of zinc (131 mg, 2.0 equiv for Entry 5; 66 mg, 1.0 equiv for Entry 6). The mixture was then stirred at room temperature for 2 h, filtered through Celite, and extracted twice with EtOAc. Next, the combined organic layers were dried over $MgSO_4$ and concentrated under reduced pressures. The residue was purified using flash chromatography on silica gel (hexane/acetone, 2:1) to yield compound **1** (178 mg, 59% for entry 5; 240 mg, 79% for entry 6).

Entry 7: AcOH (6 mL) and water (3 mL) were added to the crude reaction mixture of **7**, followed by

the addition of zinc (66 mg, 1.0 equiv). The mixture was then stirred at room temperature for 24 h, filtered through Celite, and extracted twice with EtOAc. Next, the combined organic layers were dried over $MgSO_4$ and concentrated under reduced pressures. The residue was purified using flash chromatography on silica gel (hexane/acetone, 2:1) to yield compound **1** (200 mg, 66%).

• One-pot gram-scale synthesis of cynandione A (Scheme 3 in the main text).

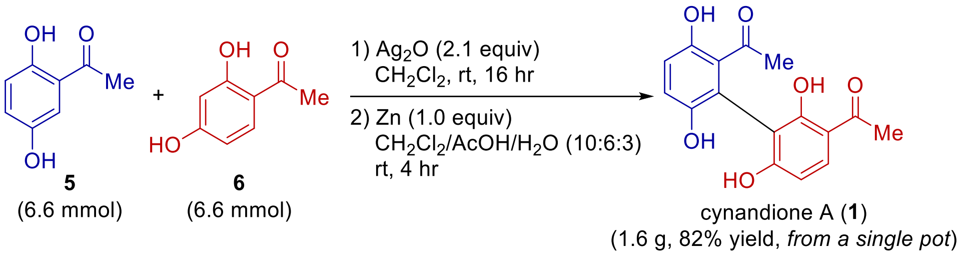

Ag_2O (3.2 g, 2.1 equiv) was added to a solution of **5** (1.0 g, 6.57 mmol, 1.0 equiv) and **6** (1.0 g, 6.57 mmol, 1.0 equiv) in CH_2Cl_2 (50 mL). The mixture was stirred at room temperature for 16 h under a nitrogen atmosphere. Next, AcOH (30 mL), water (15 mL), and zinc (430 mg, 1.0 equiv) were added to the crude reaction mixture, which was then stirred at room temperature for 4 h. The mixture was filtered through Celite, and the filtrate was diluted and extracted with EtOAc twice. The organic layer was washed with water and brine and subsequently dried over $MgSO_4$. The solvent was removed under reduced pressures, and the residue was purified by flash chromatography on silica gel (hexane/acetone, 2:1) to yield **1** (1.6 g, 82%). All spectroscopic data matched previously reported values.

^{1}H NMR (600 MHz, DMSO-d_6) δ 10.31 (s, 1H), 9.31 (s, 1H), 8.52 (s, 1H), 7.71 (d, J = 9.0 Hz, 1H), 6.74 (d, J = 8.7 Hz, 1H), 6.69 (d, J = 8.7 Hz, 1H), 6.44 (d, J = 8.9 Hz, 1H), 2.54 (s, 3H), 2.21 (s, 3H); ^{13}C{^{1}H} NMR (150 MHz, DMSO-D_6) δ 203.15, 203.06, 162.50, 162.29, 147.95, 146.93, 132.21, 130.20, 118.10, 117.34, 115.75, 112.32, 111.49, 107.48, 30.70, 26.19.

• One-pot gram-scale synthesis of cynandione A using Table 1, entry 6 conditions.

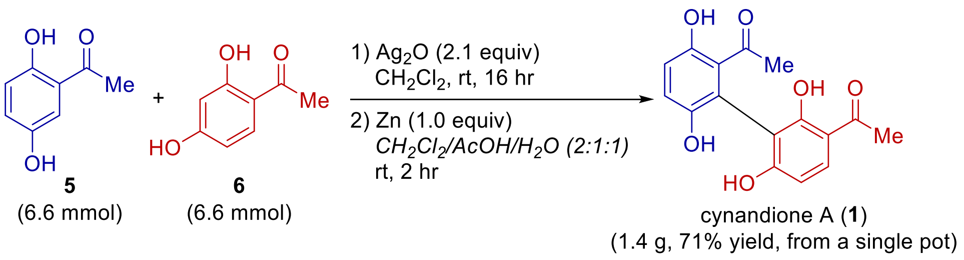

When the reaction was conducted under the same conditions but with a $CH_2Cl_2/AcOH/H_2O$ (2:1:1, 0.07 M) co-solvent system for the reduction step, compound **1** was obtained in 1.4 g with a 71% yield.

• Two-pot gram-scale synthesis of cynandione A.

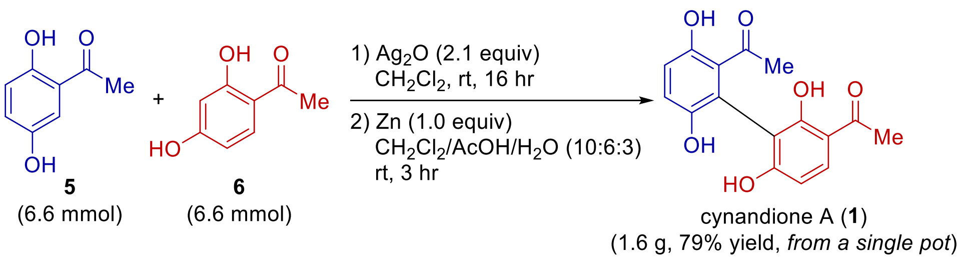

Ag_2O (3.2 g, 2.1 equiv) was added to a solution of **5** (1.0 g, 6.57 mmol, 1.0 equiv) and **6** (1.0 g, 6.57 mmol, 1.0 equiv) in CH_2Cl_2 (50 mL). The mixture was stirred at room temperature for 16 h under a nitrogen atmosphere. The reaction mixture was filtered trough a pad of Celite and concentrated under reduced pressures. The residue was dissolved with 95 mL of $CH_2Cl_2/AcOH/H_2O$ (10:6:3, 0.07 M) and zinc (430 mg, 1.0 equiv) were added to the crude reaction mixture, which was then stirred at room temperature for 3 h. The mixture was filtered through Celite, and the filtrate was diluted and extracted with EtOAc twice. The organic layer was washed with water and brine and subsequently dried over $MgSO_4$. The solvent was removed under reduced pressures, and the residue was purified by flash chromatography on silica gel (hexane/acetone, 2:1) to yield **1** (1.6 g, 79%).

3. DFT calculations

We performed DFT calculations at the M06-2X/def2-TZVP level of theory[1,2] by simulating dichloromethane with an implicit solvation model based on density (SMD) using the Gaussian 16 program package suite.[3,4] Such theoretical methods or similar levels of theory have been utilized in previous computational studies on mechanistic assessments of the synthesis of various natural products.[5-7] Geometry optimizations were conducted for all the reactants, products, and transition states, and the vibrational frequencies were calculated. The optimized structures of the reactants and products were verified by confirming the absence of imaginary frequencies. Our calculations indicated that for the TSs, only one imaginary frequency existed, and the vibrational mode of the imaginary frequency corresponded to the target reaction (conjugate addition).

Forward and reverse intrinsic reaction-coordinate calculations were performed using the TS structures to further validate the TSs and determine the appropriate conformation of the reactants and products. The aforementioned DFT calculations were repeated for multiple conformers of the reactants and products sampled using the RDKit cheminformatics package.[8] The conformers were obtained by randomly rotating the rotatable bonds and performing structural relaxation using the MMFF94 force field.[9] In addition, numerous molecular conformers were considered to identify the minimum energy paths. The percentage yields of the products (**P-3**, **P-5**, and **P-6**) were calculated from the Boltzmann weighted TSs (**TS-3**, **TS-5**, and **TS-6**) as:

$$\%\,yield\,(P-i) = \frac{\exp(-E_{TS-i}/RT)}{\sum_i \exp(-E_{TS-i}/RT)} \times 100,$$

where i = 3, 5, and 6 denote the indices of the carbons at which conjugate addition occurs. The NBO atomic charges of the molecules were calculated and analyzed using the NBO 3.0 program.[10]

Table S1. Electronic energies (E), zero-point energies (ZPE), enthalpies (H), and Gibbs free energies (G) (in Hartree, Ha) of the compounds, reactants, transition states, and products calculated in the SMD-M06-2X/def2-TZVP level of theory. The enthalpies and Gibbs free energies are calculated at 298.15 K. All the energies are in Hartree, except for ΔE_{ZPE} (kcal/mol).

Compound	E	ZPE	H	G	ΔE_{ZPE} [a,b]	imaginary frequency (cm^{-1})
**6**	-535.357546	-535.210119	-535.199200	-535.246852	N/A	
**3**	-534.101180	-533.977858	-533.967076	-534.013812	N/A	
**R'-3**	-1069.470549	-1069.199032	-1069.178355	-1069.246969	-6.9	
**R'-5**	-1069.468682	-1069.196604	-1069.174158	-1069.248974	-5.4	
**R'-6**	-1069.470546	-1069.197655	-1069.175860	-1069.246638	-6.1	
**TS-3**	-1069.438850	-1069.162886	-1069.142371	-1069.210293	15.7	-124.59
**TS-5**	-1069.429632	-1069.156974	-1069.136156	-1069.205916	19.5	-362.80
**TS-6**	-1069.402270	-1069.128607	-1069.108360	-1069.175168	37.3	-563.31
**P-3**	-1069.431361	-1069.162500	-1069.141491	-1069.211289	14.0	
**P-5**	-1069.431334	-1069.157638	-1069.136572	-1069.206984	19.0	
**P-6**	-1069.454828	-1069.177432	-1069.157355	-1069.224874	6.6	

[a] 1 Ha = 627.509391 kcal/mol. [b] Relative zero-point corrected energy with respect to **R** (**6** + **3**).

Table S2. Cartesian coordinates of the structures.

6

Atom	X	Y	Z	Atom	X	Y	Z
C	2.209532	-0.094122	-0.000018	H	-0.113139	2.866058	-0.000008
C	1.605511	-1.353126	0.000003	O	3.550536	0.061906	-0.000035
C	0.231207	-1.424874	0.000012	H	3.983244	-0.803538	-0.000005
C	-0.588998	-0.290286	0.000019	C	-2.063675	-0.520221	-0.000004
C	0.047451	0.962742	0.000033	O	-2.491345	-1.659192	0.000039
C	1.43514	1.054351	-0.000007	C	-3.024905	0.635183	-0.000085
H	2.214746	-2.248897	0.000014	H	-2.86475	1.264621	-0.875848
H	-0.25702	-2.390446	0.000011	H	-4.038205	0.240218	-0.000182
H	1.91862	2.023986	-0.000032	H	-2.864932	1.264604	0.875722
O	-0.694959	2.092975	0.000072				

3

Atom	X	Y	Z	Atom	X	Y	Z
C	-2.343834	0.608328	0.033121	C	-1.918447	-0.804955	-0.075855
C	-0.462665	-1.091909	-0.102136	O	-2.727489	-1.704579	-0.143617
C	0.443704	-0.116716	-0.036076	C	1.919093	-0.390689	-0.177122
C	-1.446483	1.585609	0.124308	O	2.548086	0.207397	-1.013828
H	-3.411271	0.791732	0.041808	C	2.518122	-1.409766	0.740353
H	-0.187479	-2.13587	-0.204041	H	3.568476	-1.556634	0.500709
H	-1.725572	2.628044	0.219958	H	2.411136	-1.06196	1.770942
C	0.009659	1.305737	0.131217	H	1.976637	-2.354607	0.663346
O	0.81105	2.194114	0.304997				

R'-3

Atom	X	Y	Z	Atom	X	Y	Z
C	2.163613	-2.119927	-0.387409	H	1.215122	2.506541	-2.561975
C	3.124633	-1.307163	0.214876	C	-1.149954	-0.997666	1.080149
C	2.98195	0.060619	0.134988	C	-2.085601	-0.313086	0.420189
C	1.921962	0.668084	-0.544134	C	-0.104753	-0.31644	1.882939
C	0.973556	-0.174385	-1.148693	H	-1.107941	-2.080822	1.051719
C	1.091324	-1.556828	-1.062436	C	-2.10472	1.177488	0.465353
H	3.964914	-1.752693	0.733279	C	-3.100435	-1.06309	-0.404059
H	3.711245	0.706726	0.606037	C	-0.10281	1.164908	1.89126

H	0.326253	-2.196564	-1.486914	O	0.711079	-0.954578	2.508918
O	-0.09143	0.371562	-1.780809	C	-1.041444	1.853562	1.246097
H	-0.635122	-0.327728	-2.173912	O	-2.950226	1.824696	-0.131324
O	2.22005	-3.467301	-0.332694	O	-2.716198	-1.996221	-1.094751
H	2.991965	-3.744917	0.180655	C	-4.541888	-0.665692	-0.310618
C	1.873602	2.160429	-0.546513	H	0.693961	1.643021	2.448536
O	2.535551	2.779686	0.264667	H	-1.068267	2.936781	1.241097
C	1.041487	2.896393	-1.558519	H	-5.16509	-1.505617	-0.61066
H	-0.019112	2.753018	-1.344124	H	-4.79417	-0.333359	0.696434
H	1.288209	3.954482	-1.508228	H	-4.715706	0.175152	-0.984824

R'-5

Atom	X	Y	Z	Atom	X	Y	Z
C	-0.381876	1.373298	-1.387782	C	1.174145	1.617418	1.351849
C	-1.472923	1.816386	-0.656481	H	3.037573	1.786064	0.206108
C	-2.448497	0.913939	-0.238584	C	1.435941	-1.196109	0.874332
C	-2.340938	-0.448481	-0.5501	C	3.670223	-0.71431	-0.410887
C	-1.221015	-0.855035	-1.288155	C	0.190556	0.660502	1.902161
C	-0.251117	0.022525	-1.715678	O	1.054417	2.814663	1.506152
H	-1.56177	2.865979	-0.393842	C	0.314537	-0.644356	1.67436
H	0.605791	-0.307301	-2.288454	O	1.491854	-2.382429	0.636205
O	0.600226	2.210161	-1.7854	O	4.139982	0.013336	-1.253681
H	0.429265	3.109474	-1.470028	C	4.27415	-2.044537	-0.079559
C	-3.311648	-1.507563	-0.145942	H	-0.628896	1.080346	2.473278
O	-3.007853	-2.677506	-0.27819	H	-0.398393	-1.373008	2.041649
C	-4.663234	-1.141651	0.400896	H	5.299834	-2.06603	-0.441513
H	-5.147795	-0.38849	-0.219711	H	4.235371	-2.242594	0.991387
H	-5.269491	-2.043107	0.45428	H	3.695528	-2.826633	-0.574243
H	-4.554035	-0.711526	1.397638	H	-1.134074	-1.908635	-1.520325
C	2.319257	1.067876	0.584171	O	-3.489626	1.351537	0.505283
C	2.462023	-0.239898	0.362432	H	-3.418408	2.307343	0.638634

R'-6

Atom	X	Y	Z	Atom	X	Y	Z
C	-0.977485	2.152879	0.670098	H	-1.311961	1.1917	2.571274
C	-0.633566	1.31404	1.735237	C	-0.373071	-0.844221	-1.391012
C	0.58802	0.677122	1.712529	C	-1.435298	-1.040939	-0.605479

C	1.491612	0.832943	0.656452	C	0.917673	-1.539017	-1.171153
C	1.089013	1.625265	-0.434454	H	-0.404717	-0.12689	-2.203265
C	-0.125469	2.299515	-0.411814	C	-1.355756	-2.050841	0.490248
H	0.884441	0.045749	2.540556	C	-2.668894	-0.199266	-0.858473
O	1.813689	1.761629	-1.571521	C	0.978054	-2.56888	-0.116626
H	2.333843	0.964406	-1.747281	O	1.90069	-1.236624	-1.821941
O	-2.15017	2.820422	0.633285	C	-0.079339	-2.79269	0.657858
H	-2.673828	2.617956	1.421788	O	-2.294094	-2.301892	1.214384
C	2.81678	0.160636	0.790657	O	-2.783146	0.344195	-1.931834
O	2.953119	-0.75355	1.578799	C	-3.700843	-0.038379	0.215917
C	4.001498	0.644109	-0.005625	H	1.91761	-3.093261	0.003025
H	3.960916	1.713062	-0.206871	H	-0.07144	-3.525321	1.455875
H	4.90572	0.395131	0.546368	H	-4.343296	0.800832	-0.040917
H	4.042989	0.10763	-0.958938	H	-4.294981	-0.952761	0.273299
H	-0.422292	2.895654	-1.264729	H	-3.241278	0.099016	1.19429

TS-3

Atom	X	Y	Z	Atom	X	Y	Z
C	-0.883738	2.331544	-0.29434	H	-1.962241	-2.822203	-1.881031
C	-2.220007	2.222587	0.014417	C	0.983526	0.79547	0.646198
C	-2.82316	0.981243	-0.106286	C	2.018686	-0.134759	0.223719
C	-2.177766	-0.176063	-0.560192	C	0.072214	0.379328	1.779203
C	-0.844318	-0.051261	-0.937752	H	1.35562	1.80641	0.804772
C	-0.101826	1.175025	-0.686018	C	1.988166	-1.501544	0.653214
H	-2.779227	3.085195	0.352098	C	3.031667	0.424248	-0.646243
H	-3.86986	0.880682	0.154419	C	-0.020469	-1.057474	2.075559
H	0.673227	1.421981	-1.41412	O	-0.570414	1.207177	2.387828
O	-0.194281	-1.060084	-1.475728	C	0.858299	-1.909228	1.548292
H	0.753494	-0.851571	-1.584619	O	2.809695	-2.375317	0.364218
O	-0.191526	3.452914	-0.214068	O	2.914027	1.584678	-1.053798
H	-0.739556	4.179861	0.126785	C	4.218531	-0.394657	-1.079077
C	-2.986451	-1.437193	-0.624453	H	-0.800115	-1.367013	2.76181
O	-4.185827	-1.370314	-0.462838	H	0.824815	-2.968414	1.778888
C	-2.308759	-2.755972	-0.84814	H	4.877822	0.236962	-1.671565
H	-1.432913	-2.858422	-0.206157	H	4.747722	-0.797896	-0.215853
H	-3.024054	-3.551135	-0.652199	H	3.894315	-1.254634	-1.666753

TS-5

Atom	X	Y	Z	Atom	X	Y	Z
C	-0.774362	2.162671	-0.260275	C	0.362921	0.153453	1.73886
C	-2.103259	2.019853	0.063457	H	1.494903	1.736863	0.799151
C	-2.716236	0.77917	-0.093326	C	2.37428	-1.511249	0.455953
C	-2.009049	-0.351097	-0.606242	C	3.236545	0.550908	-0.754189
C	-0.688988	-0.185423	-0.904137	C	0.393909	-1.298338	1.966261
C	0.022865	1.041556	-0.690342	O	-0.351227	0.891788	2.384389
H	-2.673506	2.859095	0.446284	C	1.315831	-2.050499	1.367226
H	0.802095	1.309856	-1.40304	O	3.227625	-2.313054	0.065355
O	-0.125391	3.312011	-0.160839	O	3.012436	1.711668	-1.11338
H	-0.698633	4.014697	0.186916	C	4.463663	-0.154496	-1.267935
C	-2.610326	-1.712904	-0.836633	H	-0.341153	-1.703598	2.651683
O	-1.867779	-2.66522	-0.91543	H	1.366163	-3.120514	1.535883
C	-4.092109	-1.869801	-1.01032	H	5.062404	0.558075	-1.832458
H	-4.507213	-1.071742	-1.624935	H	5.043422	-0.579003	-0.448816
H	-4.285654	-2.841734	-1.458292	H	4.176926	-0.991216	-1.906364
H	-4.57859	-1.818075	-0.034632	H	-0.130427	-1.03928	-1.271441
C	1.231345	0.694912	0.631528	O	-3.982158	0.60683	0.254245
C	2.3132	-0.116765	0.137347	H	-4.360174	1.422683	0.621948

TS-6

Atom	X	Y	Z	Atom	X	Y	Z
C	-0.93668	2.597156	-0.185666	H	0.580747	2.016306	-1.610565
C	-0.086532	1.654465	-0.84151	C	0.637852	-0.314758	0.64213
C	-0.41921	0.262278	-0.774473	C	2.022261	-0.121578	0.324041
C	-1.798185	-0.101944	-0.405614	C	0.214693	-1.725931	0.954322
C	-2.564046	0.84382	0.224144	H	0.228293	0.377945	1.37308
C	-2.104348	2.192368	0.368833	C	2.861715	-1.245898	-0.076202
H	-0.013672	-0.342072	-1.575801	C	2.429278	1.220755	0.208981
O	-3.786996	0.667786	0.757776	C	1.012679	-2.825091	0.377385
H	-4.018854	-0.266063	0.834542	O	-0.716986	-1.939988	1.697005
O	-0.559627	3.885119	-0.119306	C	2.238311	-2.597727	-0.086629
H	0.402346	3.933351	-0.233678	O	4.037538	-1.144195	-0.395837
C	-2.214576	-1.462053	-0.790337	O	1.537388	2.103523	0.378442
O	-1.401862	-2.220305	-1.292905	C	3.804933	1.658799	-0.193547
C	-3.638427	-1.922577	-0.617071	H	0.570613	-3.813378	0.408834

H	-4.359833	-1.175912	-0.951914	H	2.8648	-3.394491	-0.470252
H	-3.775873	-2.836476	-1.190188	H	3.8493	2.745908	-0.171064
H	-3.823285	-2.163919	0.435908	H	4.553901	1.238925	0.476816
H	-2.735961	2.896547	0.894827	H	4.04354	1.295306	-1.194666

P-3

Atom	X	Y	Z	Atom	X	Y	Z
C	-0.926512	2.298366	-0.253336	H	-1.968567	-2.858769	-1.851497
C	-2.272346	2.193031	0.008647	C	0.97228	0.815976	0.605688
C	-2.870615	0.951784	-0.125372	C	2.029162	-0.113142	0.159721
C	-2.215776	-0.215411	-0.545824	C	0.138702	0.407884	1.805416
C	-0.872193	-0.103555	-0.875034	H	1.365709	1.819757	0.76886
C	-0.120981	1.134831	-0.623942	C	2.09906	-1.44207	0.678447
H	-2.842627	3.058286	0.319708	C	2.97522	0.446353	-0.775852
H	-3.927148	0.856691	0.0961	C	0.149726	-1.008834	2.191793
H	0.580739	1.393063	-1.423287	O	-0.536531	1.229034	2.389084
O	-0.209066	-1.115025	-1.380908	C	1.042326	-1.845095	1.662082
H	0.754465	-0.928138	-1.392377	O	2.947426	-2.297338	0.402149
O	-0.241577	3.417144	-0.166069	O	2.809894	1.595381	-1.205597
H	-0.798913	4.15024	0.147693	C	4.15546	-0.356648	-1.256728
C	-3.033275	-1.470303	-0.632366	H	-0.569627	-1.317232	2.941399
O	-4.235976	-1.390858	-0.507017	H	1.078623	-2.887907	1.958017
C	-2.36135	-2.794979	-0.835265	H	4.745759	0.264005	-1.928414
H	-1.517073	-2.911613	-0.154658	H	4.762282	-0.692098	-0.415658
H	-3.093504	-3.582946	-0.676331	H	3.823194	-1.259164	-1.771139

P-5

Atom	X	Y	Z	Atom	X	Y	Z
C	-0.892937	2.088151	-0.160713	C	0.559284	0.359648	1.84353
C	-2.246479	1.926926	0.016616	H	1.54614	1.835002	0.674232
C	-2.806931	0.664111	-0.13832	C	2.572222	-1.323281	0.530045
C	-2.023476	-0.48739	-0.503755	C	2.996965	0.523171	-1.124466
C	-0.696795	-0.312323	-0.675216	C	0.854565	-0.989771	2.332676
C	-0.009114	0.967475	-0.507799	O	-0.224217	1.091888	2.417097
H	-2.876533	2.765476	0.290864	C	1.757019	-1.751248	1.712636
H	0.55866	1.246384	-1.409518	O	3.433421	-2.132483	0.14656
O	-0.292029	3.249351	-0.053118	O	2.662382	1.630023	-1.583844

H	-0.911247	3.9563	0.197803	C	4.144945	-0.209065	-1.773839
C	-2.588258	-1.874341	-0.701533	H	0.308397	-1.323765	3.207137
O	-1.8595	-2.819485	-0.51602	H	1.976437	-2.753018	2.066832
C	-4.001262	-2.049789	-1.172244	H	4.546556	0.415815	-2.570018
H	-4.283439	-1.279273	-1.888894	H	4.921229	-0.440749	-1.044491
H	-4.098735	-3.039925	-1.611854	H	3.81272	-1.166536	-2.176309
H	-4.676112	-1.971021	-0.3177	H	-0.080031	-1.170553	-0.92051
C	1.206803	0.805275	0.545188	O	-4.087942	0.464903	0.075015
C	2.296884	-0.042567	-0.010789	H	-4.541576	1.282368	0.344799

P-6

Atom	X	Y	Z	Atom	X	Y	Z
C	-0.754411	2.459012	0.37045	H	-0.477037	2.049694	-1.683575
C	-0.081989	1.67773	-0.73253	C	0.531788	-0.337009	0.544454
C	-0.366771	0.194674	-0.608241	C	1.963416	-0.074054	0.152761
C	-1.843468	-0.054535	-0.4211	C	0.209516	-1.764934	0.917641
C	-2.546192	0.843069	0.328775	H	0.278777	0.242812	1.439538
C	-1.944014	2.061385	0.836156	C	2.976748	-1.124926	0.259016
H	-0.047242	-0.29666	-1.525666	C	2.264334	1.09651	-0.449826
O	-3.826137	0.743085	0.719245	C	1.198012	-2.817852	0.615478
H	-4.210579	-0.115341	0.503913	O	-0.812277	-2.035489	1.511134
O	-0.161814	3.577746	0.790633	C	2.471758	-2.518981	0.364792
H	0.720438	3.648104	0.391935	O	4.181902	-0.937221	0.210111
C	-2.324603	-1.317927	-0.979006	O	1.311173	2.010269	-0.753169
O	-1.536107	-2.081834	-1.519236	C	3.607863	1.571769	-0.887964
C	-3.782892	-1.703784	-0.916963	H	0.855126	-3.841385	0.713793
H	-4.433271	-0.904948	-1.280773	H	3.223786	-3.290387	0.2426
H	-3.934869	-2.586695	-1.533285	H	3.490412	2.459759	-1.505493
H	-4.061245	-1.966245	0.109437	H	4.229641	1.809712	-0.024641
H	-2.489682	2.644103	1.565376	H	4.127242	0.794154	-1.44626

4. References

[1] Y. Zhao and D. Trulhar, *Theor. Chem. Acc.*, **2008**, *120*, 215-241.

[2] F. Weigend and R. Ahlrichs, *Phys. Chem. Chem. Phys.*, **2005**, *7*, 3297-3305.

[3] M. Frisc, G. Trucks, B. Sclegel, G. Scuseria, M. Robb, J. Ceeseman, G. Scalmani, V. Barone, G. Petersson and X. Li, Gaussian 16 (Revision C.01), Gaussian, Inc., Wallin gford, CT, USA, **2016**.

[4] A. V. Marenich, C. J. Cramer and D. G. Truhlar, *J. Phys. Chem. B*, **2009**, *113*, 6378 -6396.

[5] M. Elkin and T. R. Newhouse, *Chem. Soc. Rev.*, **2018**, *47*, 7830-7844.

[6] S. Kang, Y. Kim, S. Kim, J. Y. Ko and J. H. Kim, *Org. Biomol. Chem.*, **2023**, *21*, 1868-1871.

[7] S. Park, J. H. Kim, D. Kim, Y. Kim, S. Kim and S. Kim, *JACS Au*, **2024**, *4*, 2246-2251.

[8] S. Riniker and G. A. Landrum, *J. Chem. Inf. Model.*, **2015**, *55*, 2562-2574.

[9] T. A. Halgren, *J. Comput. Chem.*, **1996**, *17*, 490-519.

[10] A. E. Reed, L. A. Curtiss and F. Weinhold, *Chem. Rev.*, **1988**, *88*, 899-926.

5. Copies of spectra (^{1}H and ^{13}C NMR)

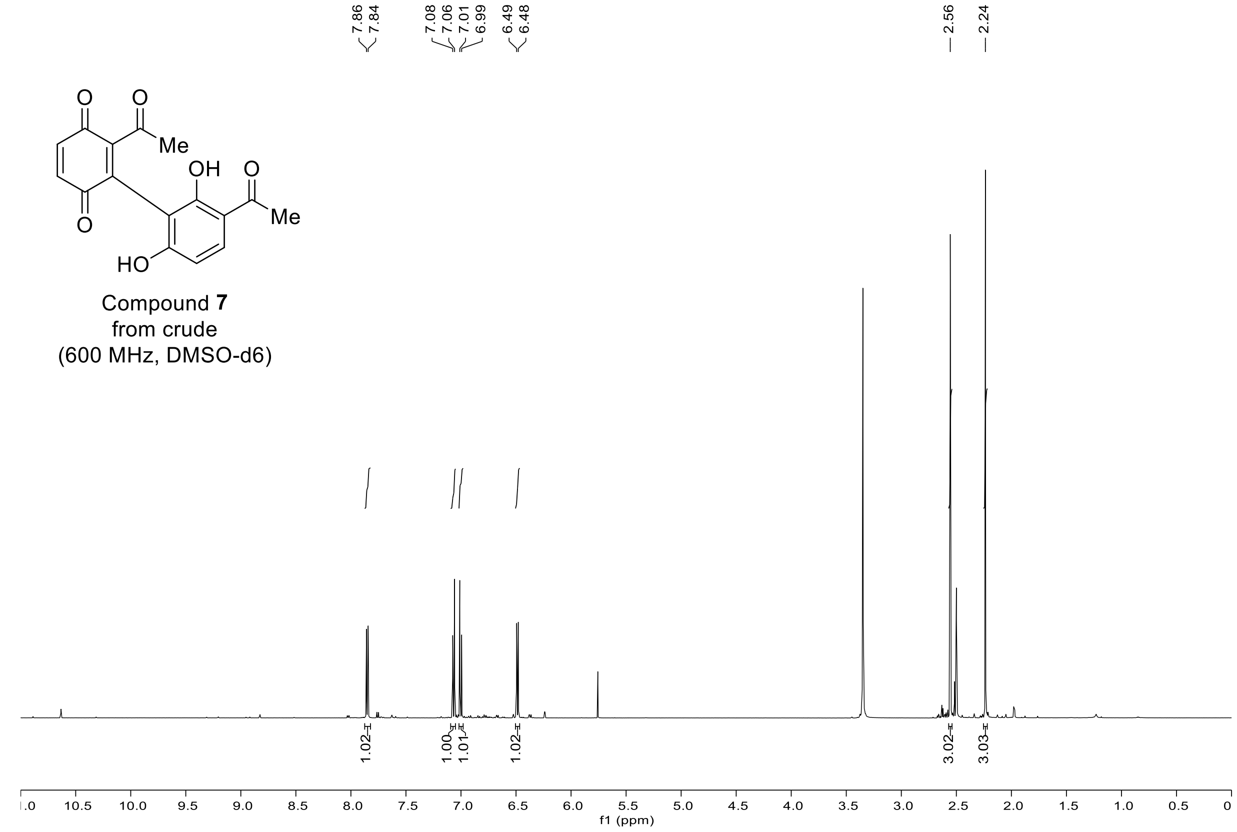

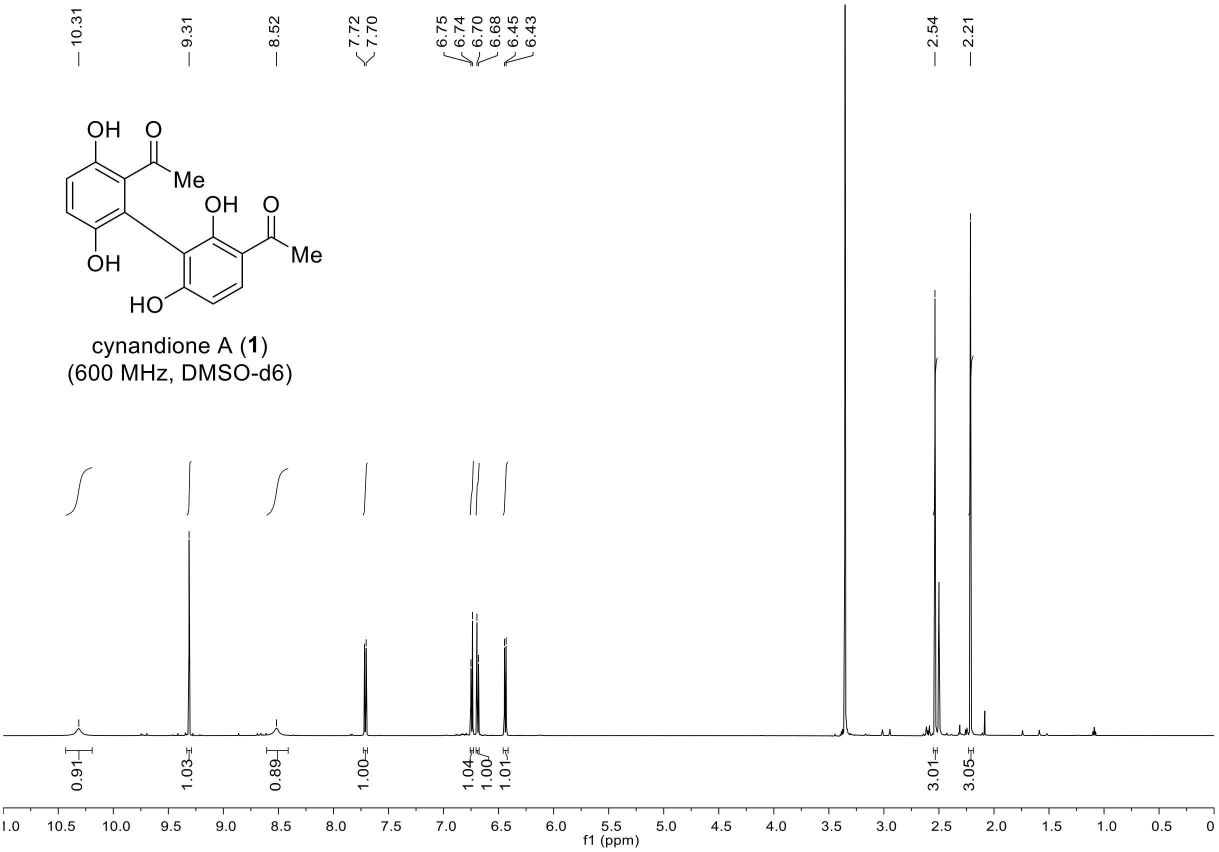

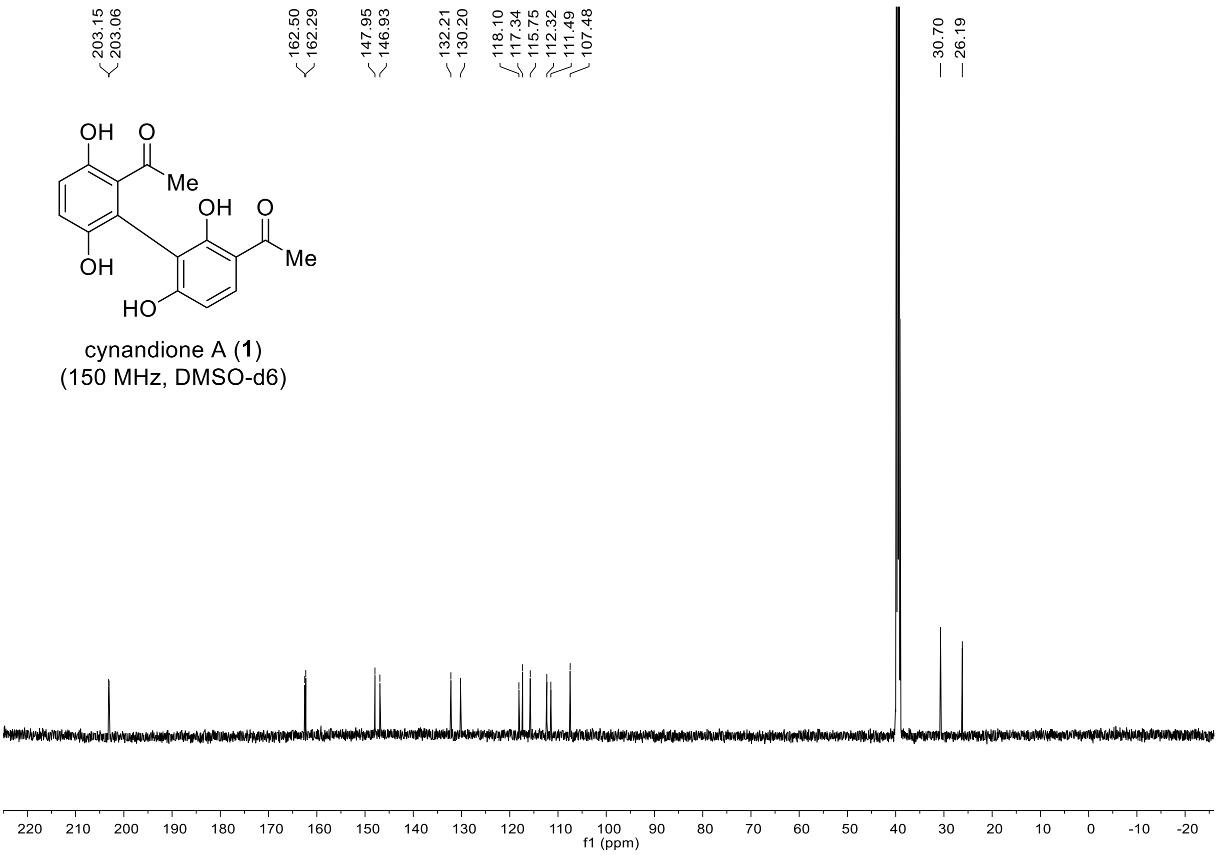

cynandione A (**1**)
(150 MHz, DMSO-d6)
